# Optimization of Polysaccharide Extraction from *Polygonatum cyrtonema* Hua by Freeze–Thaw Method Using Response Surface Methodology

**DOI:** 10.3390/molecules29204879

**Published:** 2024-10-14

**Authors:** Ziming Wang, Shushen Wu, Jiayi Wang, Ci Yang, Yang Wang, Zhan Hu, Wei Cai, Lianghong Liu

**Affiliations:** School of Pharmaceutical Sciences, Hunan Province Key Laboratory for Antibody-Based Drug and Intelligent Delivery System, Hunan University of Medicine, Huaihua 418000, China; w1026061586@gmail.com (Z.W.); wushusen1314@outlook.com (S.W.); wangjiayi810@outlook.com (J.W.); yangci1218@outlook.com (C.Y.); milk543215@outlook.com (Y.W.); hu2271727599@outlook.com (Z.H.)

**Keywords:** *Polygonatum cyrtonema* polysaccharide, freeze–thaw method, response surface methodology

## Abstract

*Polygonatum cyrtonema* polysaccharides have a variety of pharmacological effects. The commonly used extraction methods include traditional hot water extraction, alkaline extraction, enzymatic hydrolysis method, ultrasonic-assisted extraction, etc., but there are problems such as low yield, high temperature, high cost, strict extraction conditions, and insufficient environmental protection. In this study, crude polysaccharide extraction from the *Polygonatum cyrtonema* Hua was performed using the freeze–thaw method. Response surface methodology (RSM), based on a three-level, three-variable Box–Behnken design (BBD), was employed to obtain the best possible combination of water-to-raw material ratio (A: 30–50), freezing time (B: 2–10 h), and thawing temperature (C: 40–60 °C) for maximum polysaccharide extraction. Using the multiple regression analysis and analysis of variance (ANOVA), the experimental data were fitted to a second-order polynomial equation and were used to generate the mathematical model of optimization experiments. The optimum extraction conditions were as follows: a water-to-raw material ratio of 36.95:1, a freezing time of 4.8 h, and a thawing temperature of 55.99 °C. Under the optimal extraction conditions, the extraction rate of *Polygonatum cyrtonema* Hua polysaccharide (PCP) was 65.76 ± 0.32%, which is well in close agreement with the value predicted by the model, 65.92%. In addition, PCP has significant antioxidant activity. This result shows that the freeze–thaw method can improve the extraction efficiency, maintain the structural integrity of polysaccharides, simplify the extraction process, promote the dispersion of polysaccharides, and is suitable for large-scale industrial production.

## 1. Introduction

*Polygonatum cyrtonema* Hua, a perennial herb in the family Liliaceae, has been widely used as traditional Chinese medicine (TCM) and food for thousands of years in China [1,2]. Studies show that a variety of pharmacological activities have been found, such as hypoglycemia, antioxidant, immune improvement, memory improvement, antibacterial, and anti-inflammatory [3,4,5], which may be attributed to its abundant chemical ingredients: polysaccharides, steroidal saponins, triterpenes, flavones, phytosterol, volatile oils, etc. [6,7,8]. Currently, as its main active ingredients, there is a wealth of research on the extraction of Polygonatum polysaccharides, with various extraction techniques being reported, including but not limited to traditional hot water extraction, alkaline extraction, enzymatic hydrolysis method, ultrasonic-assisted extraction, microwave-assisted extraction, ultrasonic-microwave synergistic extraction, high hydrostatic pressure extraction, deep eutectic solvent method, etc. [9,10,11]. However, there are problems such as low yield rate, high temperature, high cost, strict extraction conditions, and insufficient environmental protection [12,13].

The freeze–thaw method is a technique that uses repeated freezing and melting processes to change the state and structure of substances. When the cell is frozen, ice crystals form inside the cell, which destroys the cell structure and promotes the better extraction of intracellular substances [14]. Previously, Phuong et al. used the freeze–thaw method to extract polysaccharides from spirulina. The results of the study showed that the extraction rate increased from 8.35% to 85.1% compared to the water extraction method [15]. Mylene Anwar et al. extracted taro water-soluble non-starch polysaccharide using the freeze–thaw method, which resulted in a significant increase in yield and purity [16]. Guo Weiyun et al. verified that the repeated freeze–thaw method has a destructive effect on the pollen wall, which improves the extraction rate of polysaccharides [17]. Tan Qingyun et al. not only increased the polysaccharide extraction rate from 22.5% to 41.1% by using freeze–thaw method but also better preserved the molecular structure of polysaccharides and their bioactivities [18]. Huang Yuyan et al. increased oat bran β-glucan extraction from 1.5% to 6% by using the freeze–thaw method [19]. However, no report has been used to extract *Polygonatum cyrtonem* Hua. Based on the principle that freeze–thaw can disrupt cell walls, facilitating extraction [17,20,21,22], the process was improved by mixing *P. cyrtonema* powder with pure water, freezing at low temperatures, thawing in a high-temperature water bath, and repeatedly extracting and combining the filtrate, then drying to obtain the aqueous extract. This technique is favored for its simplicity, high efficiency, low energy consumption, and excellent extraction results.

In addition, the good performance of the process was verified using the Response Surface Design of Experiments software(Design-Expert 13) box-Behnken Design (BBD) method [23]. The application of response surface methodology (RSM) in the optimization of extraction parameters is also very widespread, as it can not only analyze the mutual effects of multiple variables but also provide more intuitive and precise results [24,25,26].

In this research, the freeze–thaw method was adopted to refine *P. cyrtonema* polysaccharide (PCP). Through single-factor experiments, the effects of liquid-to-solid ratio, freezing and thawing temperatures and times, and the number of extractions on polysaccharide extraction efficiency were evaluated. Based on these experimental results, we selected the three most critical variables, and three different levels were set for each variable. The aim was to find the optimal extraction conditions through a wider combination of parameters. Subsequently, a response surface method was used for experimentation, aiming to find the optimal conditions for the extraction of PCP to maximize the extraction rate, making the process simple and efficient.

## 2. Results

### 2.1. Freeze–Thaw Method Single-Factor Experimental Results

#### 2.1.1. Liquid-Solid Ratio

**Figure 1:**
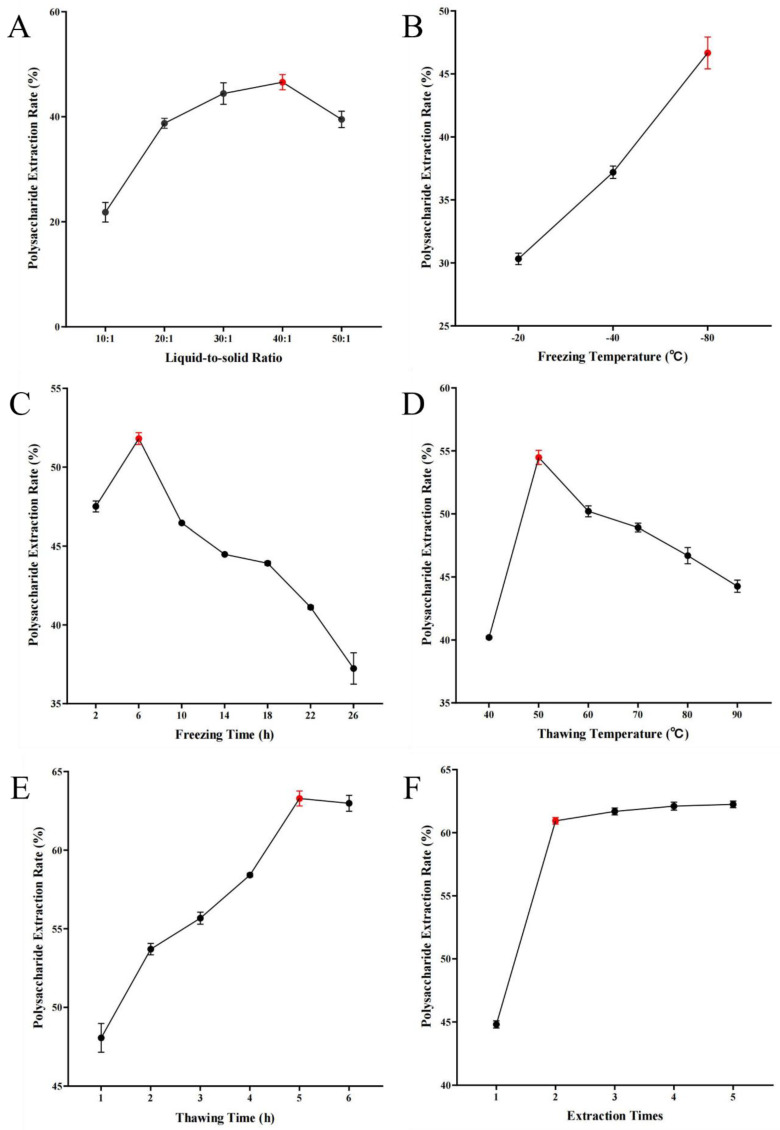
Single−factor experimental results of the freeze−thaw method: (**A**) the effect of liquid−to−solid ratio on the extraction rate of PCP; (**B**) the effect of freezing temperature on the extraction rate of PCP; (**C**) the specific effect of freezing time on the extraction efficiency of PCP; (**D**) the effect of thawing temperature on the extraction rate of PCP; (**E**) the effect of thawing time on the extraction rate of PCP; (**F**) the effect of extraction times on the extraction rate of PCP.

Figure 1A shows that as the liquid-to-solid ratio increases from 1:10 to 1:40, the extraction rate of PCP significantly increases, reaching a peak at a ratio of 1:40. This suggests that an increase in the liquid-to-solid ratio enhances the concentration gradient between the *P. cyrtonema* tissue and the solvent, thereby promoting polysaccharide diffusion [27]. When the liquid-to-solid ratio exceeds 1:40, the extraction rate decreases, possibly due to the dissolution of other components in the solution affecting the polysaccharide extraction rate [28].

#### 2.1.2. Freezing Temperature

As shown in Figure 1B, the extraction rate of PCP increases with the decrease in freezing temperature, reaching the highest point at −80 °C. This indicates that a lower freezing temperature is beneficial for improving the polysaccharide extraction rate.

#### 2.1.3. Freezing Time

Figure 1C shows that the polysaccharide extraction rate increases with the increase in freezing time, reaching the maximum value after 6 h of freezing.

#### 2.1.4. Thawing Temperature

According to the data in Figure 1D, the extraction rate of PCP significantly increases as the thawing temperature rises to 50 °C. However, once the temperature exceeds 50 °C, the extraction rate shows a downward trend. This may be because excessively high temperatures can lead to the hydrolysis of polysaccharide molecules, thereby affecting their structural integrity [29].

#### 2.1.5. Thawing Time

Figure 1E shows that as the thawing time increases, the extraction rate of PCP also increases correspondingly. When the thawing time reaches 5 h, the highest extraction rate is observed. Beyond this time duration, the extraction rate begins to decrease gradually, which may be related to the release of other components in the solution and the damage to the polysaccharide structure due to prolonged high-temperature effects [30].

#### 2.1.6. Extraction Times

Figure 1F indicates that an increase in the number of extractions has a positive impact on the extraction rate of PCP, with the rate of increase slowing down after two extractions. This may suggest that after a certain number of extractions, the improvement in extraction efficiency will tend to stabilize.

### 2.2. Freeze–Thaw Method Response Surface Experiment

#### 2.2.1. Optimization Results and Analysis of the Extraction Process

The three factors, including liquid-to-solid ratio, freezing time, and thawing temperature, were selected for further response surface analysis. Using the Design Expert 13 software, a second-order polynomial fitting was performed to construct a mathematical model with the extraction rate of PCP as the response variable. The specific experimental data and regression equation are detailed in Table 1:

R1 = 63.082 − 6.2025A − 0.785B + 4.9975C − 0.0175AB + 1.9275AC − 2.9075BC − 6.70975A^2^ − 6.94975B^2^ − 3.34475C^2^ (1)

The variance analysis results in Table 2 indicate that the established regression model is statistically significant (F = 204.7, *p* < 0.0001), and the lack of fit is not significant (F = 3.5, *p* = 0.1287, *p* > 0.05), suggesting that the model fits well and is suitable for predicting experimental results. In the model, the first-order terms A, C and their interaction term BC, as well as the second-order terms (A^2^), (B^2^), and (C^2^), all have a highly significant effect on the polysaccharide extraction rate. The AC interaction term also has a significant effect on the polysaccharide extraction rate, while the AB interaction term does not show a significant effect. Single-factor analysis shows that the main factors affecting the polysaccharide extraction rate are, in order, the liquid-to-solid ratio, thawing temperature, and freezing time.

**Figure 2:**
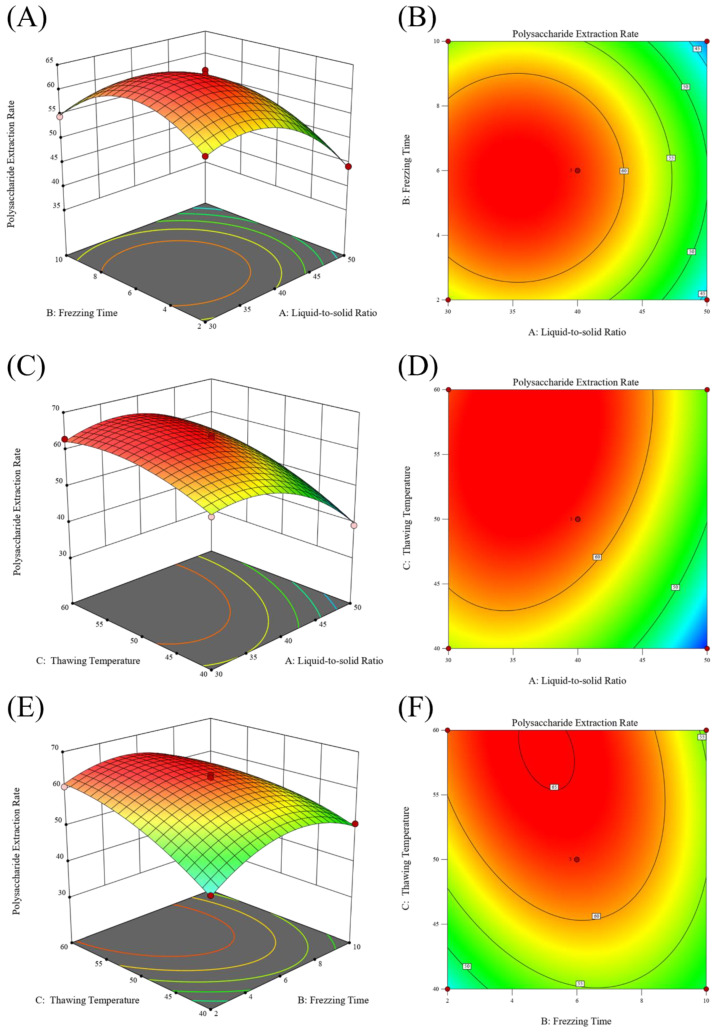
Response surface (3D) and contour plots showing the effect of the liquid−to−solid ratio, thawing temperature, and freezing time on the PCP extraction rate.

The interaction effects of various factors in the experiment are presented in the response surface and contour plots, as seen in Figure 2. Specifically, (A) and (B) represent the contour and response surface plots for the interaction between liquid-to-solid ratio and freezing time, respectively; (C) and (D) depict the contour and response surface plots for the interaction between liquid-to-solid ratio and thawing temperature; (E) and (F) illustrate the contour and response surface plots for the interaction between freezing time and thawing temperature. These plots show the combined effects of different variable combinations on the extraction rate of PCP. In the response surface plots, the steepness of the slope reflects the significance of each factor’s impact on the experiment. A steeper slope indicates a more significant impact, while a less steep slope suggests a less significant impact. The contour plots, if they approach an elliptical shape, indicate a strong interaction between the factors, whereas a circular shape suggests a weaker interaction.

#### 2.2.2. Model Adequacy Checking

**Figure 3:**
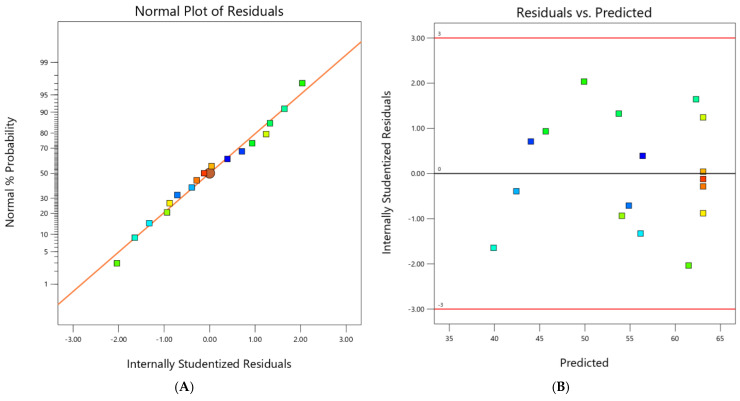
Normal probability of internally studentized residuals (**A**); plot of internally studentized residuals vs. predicted response (**B**).

It is essential to scrutinize the fitted model to ensure its adequacy in closely approximating the actual system. Should the model fail to demonstrate sufficient fit, investigating and optimizing the fitted response surface may yield poor or misleading results. The residuals from the least squares fit play a crucial role in assessing the adequacy of the model. The normality assumption is tested by constructing a normal probability plot of the residuals, as depicted in Figure 3A. The plot of the residuals approximates a straight line, satisfying the normality assumption. Figure 3B presents a plot of the residuals against the predicted responses. Visually, the residuals are randomly dispersed across the plot, indicating that the variance of the original observations is constant for all values of y. The outcomes of both plots (Figure 3) are relatively satisfactory, leading to the conclusion that the established model is adequate to describe the extraction rate of PCP using the response surface.

#### 2.2.3. Validation of the Predictive Model

Response surface optimization offers advantages over traditional one-factor-at-a-time optimization in terms of saving time, space, and raw materials. To validate the adequacy of the model equation (Equation (1)), a verification experiment was conducted under the optimal conditions (within the experimental range): liquid-to-solid ratio of 36.95:1, freezing time of 4.8 h, and thawing temperature of 55.99 °C. There must be good consistency between the predicted values from the model equation and the experimental values at the point of interest. To ensure that the predicted results do not deviate from the actual values, an experimental verification was performed using the derived optimal conditions. The set of conditions was identified as optimal through the RSM optimization method, and both experimental verification and response prediction were conducted using the model equation. The average value from the actual experiment was 65.76 ± 0.32% (n = 3), indicating the effectiveness of the RSM model. The validation results show no significant difference between the experimental and predicted values, suggesting that the response model can adequately reflect the anticipated optimization. This analysis indicates that there is a good match between the experimental and predicted values, also confirming that the model equation (Equation (1)) is satisfactory and accurate.

### 2.3. The FT-IR Spectra of PCP

**Figure 4:**
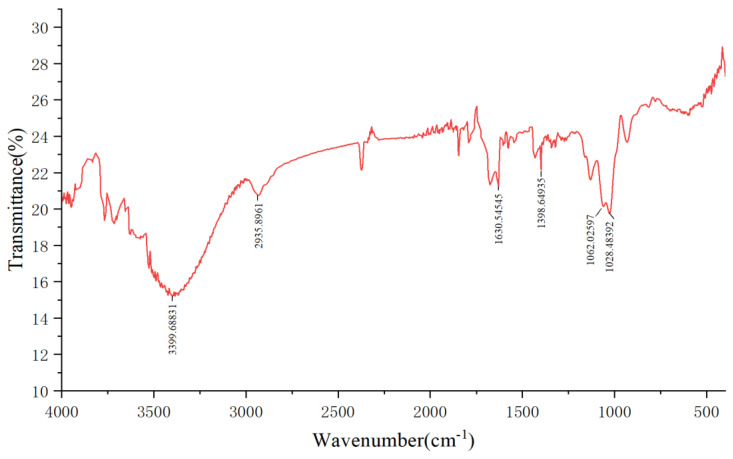
The FT−IR spectra of PCP.

Figure 4 shows the FT-IR spectra of PCP. The IR spectrogram shows that PCP has distinct polysaccharide absorption peaks [31,32]. It has absorption peaks near 3399.7 cm^−1^, 2935.9 cm^−1^, 1630.5 cm^−1^, 1398.6 cm^−1^, and 1062.0 cm^−1^. They correspond to the O-H bond, C-H bond, C=O bond, C-H bond, and C-O bond, respectively. There is also an absorption peak near 1028.5 cm^−1^, which is the characteristic absorption peak of pyranose, indicating that it contains pyranose inside [33].

### 2.4. Antioxidant Activity of PCP

#### 2.4.1. DPPH Radical Scavenging Activity

**Figure 5:**
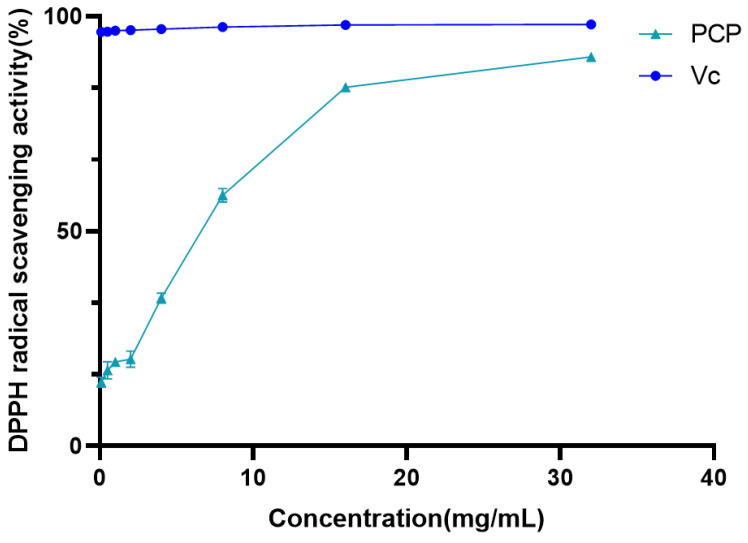
DPPH radical scavenging activity.

In the presence of antioxidants, DPPH radicals can pair with electrons or hydrogen radicals provided by antioxidants to form stable molecules. Therefore, DPPH radical scavenging assay is widely used to evaluate the activity of natural antioxidants and is recognized as the basic standard for any substance antioxidant. Through this experiment, the DPPH radical scavenging assay of PCP was measured at an absorbance of 517 nm in the concentration range of 0.1 to 32 mg/mL, and effective scavenging of DPPH radicals was obtained (Figure 5). As shown in Figure 5, within the concentration range of 0.1 to 32 mg/mL, PCP showed a significant dose-dependent relationship, with scavenging rates ranging from 14.80% to 90.49%.

#### 2.4.2. Hydroxyl Radical Scavenging Activity

Hydroxyl radicals are not only an important reactive oxygen species but also have a strong electronic oxidizing ability, which is the most toxic and harmful free radical produced during the metabolic process of living organisms. It can oxidize sugars, amino acids, proteins, nucleic acids, and other substances in tissues, leading to apoptosis or mutation.

**Figure 6:**
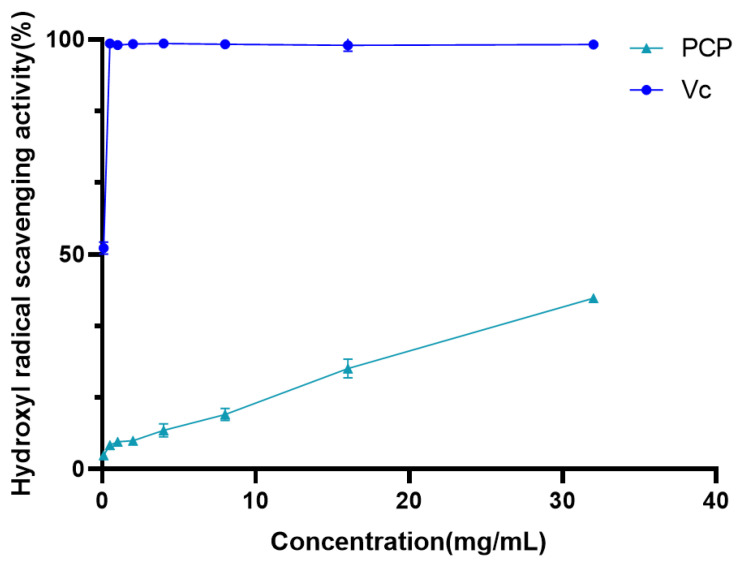
Hydroxyl radical scavenging activity.

The hydroxyl radical scavenging activity of PCP and Vc was indirectly measured using the deoxyribonucleic acid method, and the evaluation results of their hydroxyl radical scavenging ability were obtained in the range of 0.1 to 32 mg/mL (Figure 6). As shown in Figure 6, the scavenging activity of PCP increased with the increase in polysaccharide concentration, similar to the DPPH radical scavenging test. When the concentration reaches 32 mg/mL, the scavenging ability reaches 39.75%, indicating that pentachlorophenol has some hydroxyl radical scavenging activity.

## 3. Discussion

Nowadays, natural bioactive compounds are widely added to functional foods or nutritional supplements due to their anticancer properties and health benefits [34,35]. As one kind of natural bioactive compound, plant polysaccharides have antitumor, cardiovascular disease improvement, and antioxidant effects [36]. Therefore, more and more attention has been paid to extracting plant polysaccharides in this research. Prior to this, Samavati V. et al. studied the extraction process of plant polysaccharides [37,38,39]. Phytopolysaccharide extraction is gradually moving towards standardization and efficient greening. And PCP, as the main active ingredient in *Polygonatum cyrtonema* Hua, is necessary to develop new green processes. Traditional methods for extracting polysaccharides from Polygonatum, including hot water extraction, alkaline solution extraction, enzyme-assisted extraction, ultrasonic-assisted extraction, microwave-assisted extraction, the combined application of ultrasound and microwave, high-pressure technology, low-melting solvent systems, and ionic liquids combined with microwave-assisted extraction, are diverse but often face issues such as low extraction efficiency, stringent equipment requirements, and poor cost-effectiveness. Therefore, the modification of existing extraction techniques and the development of new technologies, with the aim of enhancing extraction efficiency and reducing costs, remain highly valuable areas of research. The freeze–thaw method is able to improve the extraction rate because the thermal conductivity of the cellulose that constitutes the cell wall is very low. During the freeze–thaw process, the cell wall’s inner surface temperature change is much slower than the external surface temperature change, creating a large temperature difference and thermal stress. This repeated process causes the cell wall to fracture, releasing the cell contents and increasing the polysaccharide yield [16,40,41]. This method improves extraction efficiency and quality by physically altering cell structures [14]. As an extraction technique, the freeze–thaw method has been widely applied in industrial production due to its mild operation, which is less likely to damage heat-sensitive components. It is favored for its environmental friendliness and economic benefits [22]. Moreover, its simple and environmentally friendly operational process helps to maintain the structural integrity of polysaccharides, thereby enhancing their bioactivity [17]. To date, this technology has been successfully applied to the extraction of various polysaccharides, such as those from Cornus officinalis [42], Cactaceae [20], Lycium barbarum [43], rape pollen [17,44], and Chlorella [44], all with good results. However, there are still relatively few research reports on the extraction of Polygonatum polysaccharides using this method and the optimization of its process.

In this study, the extraction process of Polygonatum polysaccharide was optimized using the response surface method. Through single-factor experiments, the appropriate range of levels for each influencing factor was determined, and the construction of the response surface and data analysis were completed using the Design-Expert software. This process helps to accurately adjust the extraction conditions to achieve the best extraction results. A single-factor investigation was conducted on six aspects: liquid-to-solid ratio, freezing temperature, freezing time, thawing temperature, thawing time, and the number of extraction cycles. As the liquid-to-solid ratio increased, the concentration difference between the Polygonatum tissue and the solvent increased, promoting polysaccharide diffusion [27], reaching an optimal state at a ratio of 1:40. Within a certain range, the polysaccharide extraction rate increased significantly with the extension of freezing time, especially after 6 h, which may be due to the complete destruction of the three-dimensional reticular structure of plant tissue cell walls over time, facilitating the complete release of the polysaccharide [42]. Within a certain range, the polysaccharide extraction rate first increased and then decreased with the increase in thawing temperature, peaking at 50 °C. Although increasing the thawing temperature generally favors the dissolution of the extractable components from the plant, excessively high thawing temperatures may lead to uneven internal temperature distribution during sample thawing, thereby increasing mass transfer resistance and being unfavorable for the dissolution kinetics of the extractable components. Another possibility is that temperatures exceeding 50 °C may accelerate the decomposition of a small amount of heat-unstable polysaccharides [43]. Additionally, within a certain range, the polysaccharide content gradually increased with the number of freeze–thaw cycles, but the increase was not significant after more than two cycles. This may be because the cell wall structure was essentially destroyed after multiple freeze–thaw cycles [42]. Finally, based on the results of the single-factor experiments, three factors, namely liquid-to-solid ratio, freezing time, and thawing temperature, were selected, and the freeze–thaw extraction process for Polygonatum polysaccharide was finely adjusted using the response surface analysis method. The optimal extraction conditions obtained were a liquid-to-solid ratio of 36.95:1, a freezing period of 4.8 h, and a thawing process temperature controlled at 55.99 °C, under which the polysaccharide extraction rate could reach 65.92%. To facilitate practical operation, the final extraction process was set to a liquid-to-solid ratio of 40:1; low-temperature freezing at −80 °C for 5 h; and thawing at 56 °C for 5 h, repeated twice, and the combined filtrate yielded a polysaccharide extraction rate of 65.76%. Compared with the traditional water extraction method of a liquid-to-solid ratio of 1:20, with three extractions at 80 °C water bath, which resulted in an extraction rate of 33.87%, there was a significant improvement. When compared with the traditional water extraction–alcohol precipitation method and ultrasonic extraction method, the latter showed higher extraction efficiency, simpler operation, lower energy consumption, and reduced time cost, making it more suitable for the extraction of Polygonatum polysaccharide. According to the data obtained by D. Chu [45] using the response surface method, the yield of Polygonatum polysaccharide by the traditional water extraction method was 12.50%. While Z. Du [46] achieved an extraction rate of 14.09% for *Polygonatum kingianum* under optimized ultrasonic extraction conditions. In comparison, the polysaccharide extraction rate obtained in this study was higher, indicating that the optimized extraction process is not only simple but also efficient. In addition, the infrared absorption spectrum of PCP exhibited characteristic absorption peaks of polysaccharides. In the DPPH radical scavenging assay, the DPPH radical scavenging activity of PCP increased rapidly with increasing concentration, and the scavenging rate reached 90.49% when the concentration of PCP reached 32 mg/mL. However, the increase in DPPH radical inhibition gradually leveled off in the range of 16 to 32 mg/mL. In the hydroxyl radical scavenging assay, the hydroxyl radical scavenging activity of PCP showed a dose-dependent relationship. The scavenging efficiency reached 39.75% when the content of PCP reached 32 mg/mL. The results of the antioxidant test showed that PCP has some antioxidant activity. This lays a solid foundation for the further separation, extraction, and application development of Polygonatum polysaccharides.

## 4. Materials and Methods

### 4.1. Reagents and Materials

Chemical reagents were received from commercial sources (Guangzhou Watson’s Food & Beverage Co., Ltd. (Haizhu District, Guangzhou, China), Chongqing Chuandong Chemicals (Nananqu District, Chongqing, China)). *P. cyrtonema* Hua was purchased from Hunan Boshikang Traditional Chinese Medicine Co., Ltd. (Huaihua, Hunan, China)

### 4.2. P. cyrtonema Hua Material Pretreatment and Polysaccharide Extraction

An amount of 10 kg of *P. cyrtonema* Hua was taken, dried, ground, and sifted by an 80 mesh sieve to obtain the *P. cyrtonema* Hua powder.

The *P. cyrtonema* Hua powder was at a certain liquid-to-solid ratio; water was added and vortexed for 30 s. The extraction liquid was placed in the refrigerator to freeze for a certain period, then taken out and placed into a water bath pot at a certain temperature for high-temperature thawing. After thawing for a certain period, the above operation (freezing, placement, and thawing) was repeated a certain number of times, the filtrate was combined, and the *P. cyrtonema* Hua aqueous extract was obtained.

### 4.3. Determination of Sugar Content

**Figure 7:**
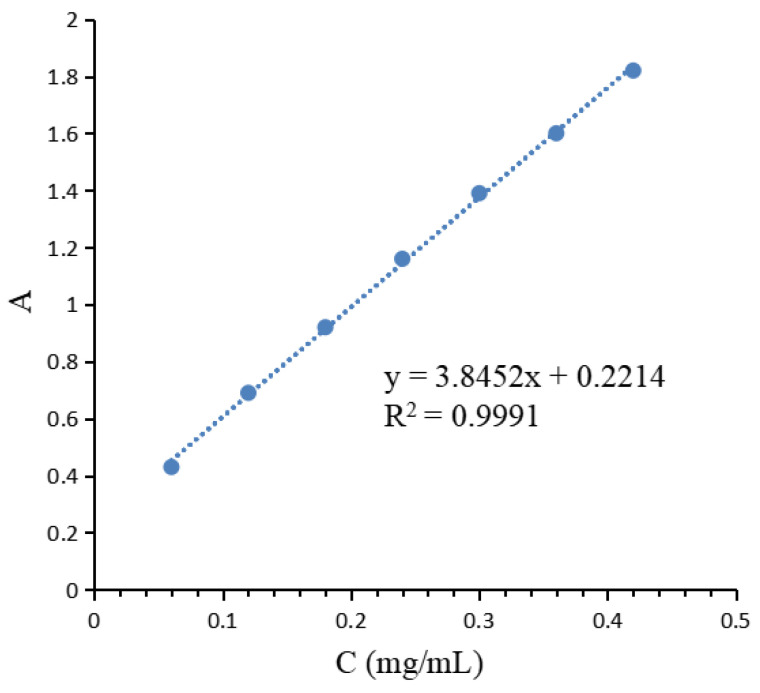
Standard curve of glucose.

The sugar content in the extract was determined using the sulfuric acid–phenol method [47,48]. An amount of 0.06 g of anhydrous glucose was accurately weighed out and completely dissolved in an appropriate amount of distilled water, and the solution was then transferred to a 100 mL volumetric flask (0.6 mg/mL). Amounts of 0, 0.1, 0.2, 0.3, 0.4, 0.5, 0.6, and 0.7 mL of the 0.6 mg/mL anhydrous glucose solution were accurately pipetted into stoppered test tubes, to which 1.0, 0.9, 0.8, 0.7, 0.6, 0.5, 0.4, and 0.3 mL of deionized water were added, respectively. Then, 0.5 mL of freshly prepared 5% phenol solution was rapidly added to each tube. After thorough mixing, 2.5 mL of concentrated sulfuric acid was quickly added; the tubes were immediately capped, mixed well, and then heated in a water bath at 90 °C for 15 min before cooling to room temperature. The absorbance was measured at 490 nm using a microplate reader, and a standard curve was constructed. The standard curve was plotted with mass concentration (mg/mL) on the x-axis and absorbance on the y-axis, as shown in Figure 7.

After adding 1 mL of the sample solution to a stoppered test tube, 1 mL of freshly prepared 5% phenol solution was rapidly added. The mixture was thoroughly mixed, followed by the rapid addition of 5 mL of concentrated sulfuric acid, capping, and mixing. The test tube was then placed in a water bath at 90 °C for 15 min before cooling to room temperature. The absorbance was measured at 490 nm using a spectrophotometer. The sugar content in the extract was calculated using the formula derived from the standard curve, and the mass of sugar in the extract was further determined based on the volume of the extract. The polysaccharide extraction rate was then calculated using the following Formula (2):

(2) $$Polysaccharide\;Extraction\;Rat=\frac{Mass\;of\;Sugar\;in\;the\;Extract\;(m)}{Mass\;of\;Polygonatum\;Powder\;(M)}\times 100\%$$

m is the Glu mass of sugar in extract (g), and M is the mass of Polygonatum powder (g)

### 4.4. Single-Factor Experiments for Polysaccharide Extraction

#### 4.4.1. Effect of Liquid-to-Solid Ratio on the Extraction Rate of PCP

With the conditions of freezing time at 2 h, freezing temperature at −80 °C, thawing temperature at 80 °C, thawing time at 2 h, and extraction times set to 3 times held constant, different liquid-to-solid ratios (10:1, 20:1, 30:1, 40:1, 50:1) were set to assess their specific impact on the polysaccharide extraction rate.

#### 4.4.2. Effect of Freezing Temperature on the Extraction Rate of PCP

With the liquid-to-solid ratio fixed at 40:1, freezing time at 2 h, thawing temperature at 80 °C, thawing time at 2 h, and extraction times at 3 times, the freezing temperatures were designed to be −20, −40, and −80 °C for the experiment.

#### 4.4.3. Effect of Freezing Time on the Extraction Rate of PCP

With the liquid-to-solid ratio fixed at 40:1, freezing temperature at −80 °C, thawing temperature at 80 °C, thawing time at 2 h, and extraction times at 3 times, the freezing times were designed to be 2, 6, 10, 14, 18, 22, and 26 h for the experiment.

#### 4.4.4. Effect of Thawing Temperature on the Extraction Rate of PCP

With the liquid-to-solid ratio fixed at 40:1, freezing temperature at −80 °C, freezing time at 6 h, thawing time at 2 h, and extraction times at 3 times, the thawing temperatures were designed to be 40, 50, 60, 70, 80, and 90 °C for the experiment.

#### 4.4.5. Effect of Thawing Time on the Extraction Rate of PCP

Under the fixed conditions of a liquid-to-solid ratio of 40:1, freezing temperature controlled at −80 °C, freezing time set to 6 h, thawing temperature maintained at 50 °C, and extraction times at 3 times, this study explored the specific impact of thawing time (1 to 6 h) on the polysaccharide extraction efficiency.

#### 4.4.6. Effect of Extraction Times on the Extraction Rate of PCP

Maintaining the liquid-to-solid ratio at 40:1, with the freezing temperature at −80 °C, freezing time at 6 h, thawing temperature at 50 °C, and thawing time at 5 h, the extraction times were designed to be 1, 2, 3, 4, and 5 times for the experiment.

### 4.5. Response Surface Experiment for Polysaccharide Extraction

After integrating the findings of the single-factor experiments and the basic principles of the response surface method, this study designed an optimization experiment for the extraction efficiency of PCP, with the liquid-to-solid ratio, freezing time, and thawing temperature as independent variables. The related factors and their levels are detailed in Table 3.

### 4.6. FT-IR Spectrometric Analysis

PCP was analyzed using Fourier Transform Infrared Spectroscopy (IRAffinity-1S). Firstly, about 1 mg of dried PCP was weighed and ground into very small particles, which were then ground together with about 100 mg of KBr powder and pressed into a transparent circular flake of about 1 mm to serve as a test sample. The scanning range was 4000 cm^−1^ to 400 cm^−1^.

### 4.7. Determination of Antioxidant Activity of PCP

#### 4.7.1. DPPH Radical Scavenging Activity

For this experiment, 2.5 mL of 100 μmol/L DPPH ethanol solution was added to 2 mL of polysaccharide solutions of different concentrations, stored in the absence of light for 30 min, and then the absorbance (A1) was measured at 517 nm using deionized water [49,50]. The absorbance (A0) of a solution treated with deionized water instead of a polysaccharide solution was determined. The absorbance of a solution using deionized water instead of DPPH ethanol solution (A2) was measured.

DPPH radical scavenging activity (%) = (A0 − A1 + A2)/A0 × 100 (3)

#### 4.7.2. Hydroxyl Radical Scavenging Activity

For this experiment, 1 mL of polysaccharide solutions of different concentrations were added to 1.0 mL of 9 mmol/L FeSO_4_, 1.0 mL of 9 mmol/L salicylic acid, and 0.5 mL of 0.1% H_2_O_2_ at 37 °C, and stored for 30 min [51]. The absorbance of the obtained suspension at 510 nm (A1) was measured using deionized water as a reference. This solution, which uses deionized water instead of polysaccharide solution, was treated with the same method to determine its absorbance (A0). An amount of 1.0 mL polysaccharide solutions of different concentrations was added to 2.5 mL deionized water to measure their absorbance (A2). Vc serves as a positive control for all these tests.

Hydroxyl radical scavenging activity (I%) = (A0 − A1 + A2)/A0 × 100 (4)

## 5. Conclusions

The present study successfully optimized the extraction process of PCP using the freeze–thaw method and response surface methodology (RSM). By combining the results of the single-factor experiments, the optimal extraction process parameters were established as follows: a liquid-to-solid ratio of 36.95:1, a freezing temperature of −80 °C, a freezing time of 4.8 h, a thawing temperature of 55.99 °C, and a thawing time of 6 h. With two extraction times, the polysaccharide extraction rate was 65.76%, which is in excellent concordance with the predicted model value of 65.92%. Meanwhile, the antioxidant experiments showed that PCP has significant antioxidant activity. This study applies the freeze–thaw method as an innovative strategy for the efficient extraction of PCP, laying a solid foundation for further research on its separation, purification, and application in the pharmaceutical and nutraceutical industries. In the future, further work will be carried out to purify the crude extract in order to better characterize PCP and determine its pharmacological activity through in vitro and/or in vivo experiments.

## Figures and Tables

**Table 1:** Response surface optimization design and results.

No.	A: Liquid-to-Solid Ratio	B: Freezing Time (h)	C: Thawing Temperature (°C)	Polysaccharide Extraction Rate (%)
1	40	6	50	62.49
2	40	6	50	63.00
3	30	6	40	55.66
4	40	10	40	50.68
5	40	2	40	46.02
6	40	10	60	53.74
7	40	2	60	60.71
8	50	6	40	39.28
9	50	6	60	54.25
10	50	10	50	42.27
11	40	6	50	62.89
12	40	6	50	63.92
13	50	2	50	44.29
14	30	2	50	56.54
15	30	6	60	62.92
16	40	6	50	63.11
17	30	10	50	54.59

**Table 2:** Variance and significance analysis of the response surface quadratic regression equation.

Source of Variation	Sum of Squares	Degrees of Freedom	Mean Square	F Value	*p* Value	Significance
Model	1047.24	9	116.36	204.7	<0.0001	***
A—Liquid-to-solid Ratio	307.77	1	307.77	541.42	<0.0001	***
B—Freezing Time	4.93	1	4.93	8.67	0.0216	*
C—Thawing Temperature	199.8	1	199.8	351.48	<0.0001	***
AB	0.0012	1	0.0012	0.0022	0.9643	
AC	14.86	1	14.86	26.14	0.0014	**
BC	33.81	1	33.81	59.49	0.0001	**
A^2^	189.56	1	189.56	333.47	<0.0001	***
B^2^	203.36	1	203.36	357.75	<0.0001	***
C^2^	47.1	1	47.1	82.87	<0.0001	***
Residual	3.98	7	0.5684			
Lack of Fit	2.88	3	0.9607	3.5	0.1287	
Pure Error	1.1	4	0.2743			
Total	1051.22	16				

Note: * *p* < 0.05 indicates significance; ** *p* < 0.01 indicates highly significant; *** *p* < 0.001 indicates very significant.

**Table 3:** Response surface analysis experimental design and factor levels for freeze–thaw extraction of PCP.

Factor	A: Liquid-to-Solid Ratio	B: Freezing Time (h)	C: Thawing Temperature (°C)
−1	30:1	2	40
0	40:1	6	50
1	50:1	10	60

## Data Availability

The data presented in this study are available on request from the corresponding author.
